# Application of a long short-term memory neural network algorithm fused with Kalman filter in UWB indoor positioning

**DOI:** 10.1038/s41598-024-52464-y

**Published:** 2024-01-22

**Authors:** Yalin Tian, Zengzeng Lian, Penghui Wang, Mengqi Wang, Zhe Yue, Huabin Chai

**Affiliations:** School of Surveying and Land Information Engineering, Henan Polytechnic University, Jiaozuo, 454003 China

**Keywords:** Engineering, Mathematics and computing

## Abstract

Ultra-wideband technology has good anti-interference capabilities and development prospects in indoor positioning. Since ultra-wideband will be affected by random errors in indoor positioning, to exploit the advantages of the Kalman filter (KF) and the long short-term memory (LSTM) network, this paper proposes a long short-term memory neural network algorithm fused with the Kalman filter (KF–LSTM) to improve UWB positioning. First, the ultra-wideband data is processed through KF to weaken the noise in the data, and then the data is fed into the LSTM network for training, and the capability of the LSTM network to process time series features is employed to obtain more accurate label positions. Finally, simulation and measurement results show that the KF–LSTM algorithm achieves 71.31%, 37.28%, and 49.31% higher average positioning accuracy than the back propagation (BP) network, (back propagation network fused with the Kalman filter (KF-BP), and LSTM network algorithms, respectively, and the KF–LSTM algorithm performs more stably. Meanwhile, the more noise the data contains, the more obvious the stability contrast between the four algorithms.

## Introduction

In today’s digital era, technology is developing rapidly, and numerous intelligent fields have increasingly higher requirements for location accuracy. In outdoor environments, GNSS (Global Navigation Satellite System) technology can provide accurate location services. Nevertheless, due to the obstruction of buildings and walls, GNSS signals cannot directly reach indoors, limiting the application of GNSS technology in indoor environments^1,2^. Therefore, some indoor intelligent applications based on location services have urgent needs for precise positioning within indoor settings, such as airports and large retail centers. In recent years, various indoor positioning technologies have emerged, including Wi-Fi fingerprint^3^, Bluetooth^4^, UWB (Ultra-Wideband)^5,6^, Zigbee^7^, ultrasonic^8^, audio^9^ positioning technologies, etc. Among them, UWB positioning technology is commonly utilized in indoor positioning because of its high accuracy, low power consumption, and anti-interference characteristics^10^. Many UWB positioning algorithms have been proposed, such as TOA(Time of Arrival) positioning and TDOA(Time Difference of Arrival) positioning, which rely on UWB equipment for ranging and finding the tag position based on related algorithms^11,12^, wireless signal arrival angle AOA (Angle of Arrival) positioning^13^ and fingerprint matching positioning based on signal strength RSSI (Received Signal Strength Indication)^14^. However, due to the high complexity of the indoor environment, the signal will be affected by factors such as multipath, occlusion, noise interference, and signal attenuation in the transmission process, which increases the error of UWB indoor positioning, making positioning results fail to meet the requirements^15^. Thus, new algorithms need to be developed to minimize the influence of these factors on positioning accuracy.

Kalman filter is a recursive algorithm for estimating system state, and it performs well when processing measurement data containing noise and uncertainty^16^. The indoor environment is complex, and UWB will inevitably be interfered with by WIFI, Bluetooth, and other signals, making acquired data contain a certain amount of noise. Combining Kalman filtering and UWB technology in indoor positioning can make the positioning results more accurate. Cheng et al. developed a positioning system that combines UWB technology and the extended Kalman filter algorithm to enhance positioning precision^17^. However, the system was only compared with the TOA algorithm, not other algorithms, and it was not tested in practical application scenarios.

Deep learning has powerful pattern recognition and learning capabilities, and it has achieved great success in many fields, such as sensor fusion, signal processing, feature extraction, etc.^18–20^. Deep learning can also assist UWB positioning by using its unique learning capabilities to learn key features from signals, such as delay, amplitude, multipath, etc.^21^. Doan Tan Anh Nguyen developed an ultra-wideband positioning method based on convolutional neural networks, which uses two-dimensional images with three channels to calculate the location of tags. This method avoids the problems of error propagation and the high complexity of traditional methods, and the performance of the method in different channel models and regional environments was verified through simulation experiments^22^. Alwin Poulose et al. used UWB distance information to train the LSTM model, analyzed the impact of batch size, optimizer, learning rate, time steps, and the number of hidden nodes and loss function on the performance of the LSTM model, and a parameter model was constructed to improve UWB positioning accuracy^23^. However, the research on UWB positioning was only conducted theoretically based on simulation experiments, and there was no verification in practical applications. Gao et al. proposed using the LSTM network to predict the ranging error between the anchor point and the target, and they combined weighted least squares and regression weighted least squares for positioning correction, which improved UWB positioning accuracy^24^. However, their study did not take other deep learning algorithms for comparison and did not analyze the limitations and advantages of the LSTM algorithm in UWB indoor positioning systems.

When UWB is used for indoor positioning, it can be affected by various interference sources indoors, as well as multipath interference generated by UWB signals indoors. These interferences introduce a significant amount of random noise into UWB ranging information, subsequently affecting the positioning results of UWB. To mitigate the impact of random errors on UWB indoor positioning, this paper combines the strengths of KF and LSTM, proposing a long short-term memory network fused with the Kalman filter (KF–LSTM). The algorithm utilizes Kalman filtering to handle Gaussian noise in UWB ranging time series and combines the advantages of LSTM networks in time series analysis to achieve accurate tag positioning. The rest of this paper is organized as follows: In “Related work” section introduces related work, mainly including the research status and challenges of UWB. In “UWB ranging principle” section describes the principle of UWB ranging. In “UWB positioning based on the KF–LSTM algorithm” section provides the principle of the KF–LSTM algorithm. In “Data simulation and result analysis” and “Measured data and result analysis” section analyzes the simulation and measured experimental results. In “Discussion” section is the discussion part. In “Conclusions” section concludes this paper.

## Related work

### UWB positioning based on Kalman filter

Kalman filtering is an excellent state estimation method that can effectively weaken the noise of UWB signals and the uncertainty in state estimation, thereby achieving more accurate positioning results. In the literature^25^, a tracking algorithm based on the Kalman filter was proposed to enhance the positioning accuracy in the NLOS environment. In the literature^26^, the NLOS environment was studied again, and an improved incremental Kalman filter was proposed based on the work in^25^, which further improved the positioning accuracy. However, if the indoor space is narrow, its positioning accuracy and stability will be significantly reduced. In the literature^27^, a narrow space ultra-wideband positioning algorithm combining a precision dilution mathematical model and the Kalman filter was proposed. With the use of this technique, positioning stability and precision can be increased while also successfully suppressing positioning errors brought on by small space structures. However, this algorithm cannot solve the positioning problem in nonlinear systems, and solutions are given in the literature^28,29^. In^28^, an unscented Kalman filter algorithm based on the maximum correlation entropy criterion was proposed. In this algorithm, the prediction state estimate and covariance matrix are obtained through unscented transformation, and the observation data is rebuilt using the nonlinear regression approach based on the maximum correlation entropy criterion to enhance the unscented Kalman filter’s placement accuracy in a non-Gaussian noise environment. In^29^, a robust extended Kalman filter algorithm was proposed, which can resist outliers in observation data and enhance the accuracy and reliability of ultra-wideband positioning. However, the effects of dynamic model errors and observation system errors were not considered. In the literature^30^, an improved robust adaptive cubic Kalman filter algorithm was proposed to deal with the non-line-of-sight error and motion model error of UWB positioning, a polynomial fitting sliding window was designed to determine the error type of signal propagation in real time, and a correction plan was given to improve UWB positioning accuracy.

### UWB positioning based on deep learning

As a branch of machine learning, deep learning can imitate the human brain for learning and pattern recognition. When applied to UWB positioning systems, it can perform large-scale data processing and extract non-linear relationships from the data. In the literature^31^, a CNN data model based on the received signal strength fingerprint was proposed and compared with deep learning models such as AlexNet and ResNet. The results indicate that the proposed CNN model has higher test accuracy than other deep learning models. However, when using RSSI for positioning, the amount of data required to be collected is large, and the final positioning accuracy is not as good as that using ranging. In the literature^32^, a CNN-based UWB positioning method was proposed, which uses CNN to replace TOA for distance estimation to improve positioning accuracy. However, this study only took TOA for comparison without considering other deep learning models. In the literature^33^, it was proposed to transmit UWB signals to three different receiving antenna devices and convert the signals into RGB images. By using a CNN model to process the received image and estimate the location of the label, this method does not require predicting the distance between the transmitter and the receiver. The simulation results demonstrate that the performance is significantly improved compared with the previous CNN positioning method. However, when positioning, the transmission signal needs to be converted into an RGB image for processing, which increases the calculation time and has a certain impact on real-time positioning. In the literature^34,35^, the advantage of high ranging accuracy of UWB was exploited to combine UWB and inertial navigation systems, and the LSTM network was used to process the time series in the position estimation process, thereby achieving higher positioning accuracy. However, this solution needs to be combined with the INS system, which will increase the cost of positioning. In the literature^36^, CNN and LSTM were combined to use RSS and distance data to estimate the tag position, exploit the feature extraction ability of CNN to suppress measurement noise, and adopt the LSTM model to establish the correlation of consecutive frames, thereby enhancing positioning accuracy.

In related work, the current research status of UWB is introduced, and the challenges encountered by UWB in the development of indoor positioning and different methods proposed for UWB are explained. Although many scholars have achieved good results in experiments, they still need to be verified in other environments, and the UWB indoor positioning task still has a long way to go. This paper proposes the KF–LSTM algorithm by combining Kalman filtering and the LSTM neural network. Analysis of simulation and measured data shows that the KF–LSTM algorithm achieves good indoor positioning accuracy and increases the precision and stability of UWB positioning.

## UWB ranging principle

**Figure 1:**
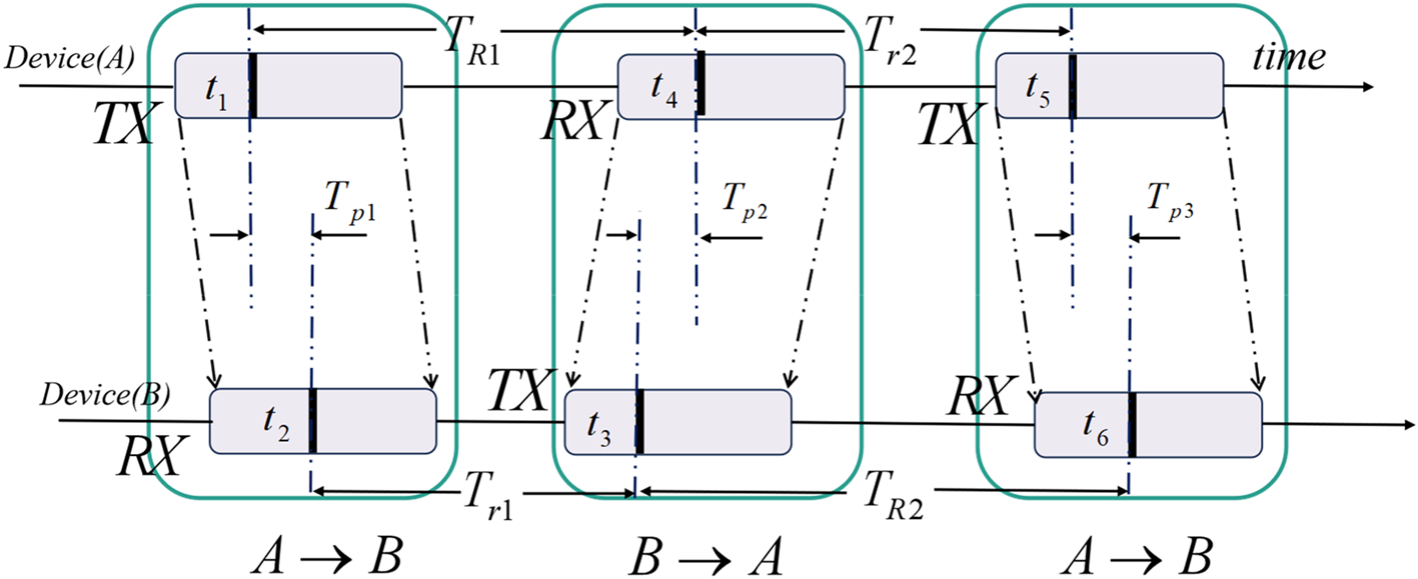
UWB ranging principle.

UWB technology is a high-rate communication and high-precision positioning technology that uses nanosecond-level non-sinusoidal narrow pulses for data transmission. Its operating frequency band is 3.1 GHZ ~ 4.8 GHZ, ensuring sub-nanometer precise time. The short pulse width of the UWB time domain signal results in enhanced range and locating accuracy due to its increased time and space resolution. In UWB positioning technology, double-side two-way ranging (DS-TWR) principle is a commonly used method to achieve high-precision distance measurement by measuring the round-trip propagation time of signals, as shown in Fig. 1:

In Fig. 1, $t_{1}$, $t_{4}$ and $t_{5}$ are moments recorded by device A. $t_{2}$, $t_{3}$ and $t_{6}$ are moments recorded by device B.$T_{p1}$ indicates the time the signal traveled from device A to device B. $T_{p2}$ indicates the time the signal returned from device B to device A. $T_{p3}$ indicates the time the signal traveled from device A to device B for the second time. Since the time interval between the two signal transmissions is very short, it can be assumed that the distance from device A to device B has not changed, so $T_{p1}$, $T_{p2}$ and $T_{p3}$ are theoretically equal. In the following equations, we use $\hat{T}_{p}$ instead of the time it takes for the signal to travel from device A to device B.$T_{R1}$ denotes the interval between device A sending information and device B receiving the response.$T_{R2}$ denotes the interval between device B sending information and device A receiving response.$T_{r1}$ denotes the response time for device B to process device A’s information. $T_{r2}$ denotes the response time for device A to process device B’s information. The following equation can be obtained:

1 $$ \left\{ {\begin{array}{*{20}c} {T_{R1} = t_{4} - t_{1} } \\ {T_{R2} = t_{6} - t_{3} } \\ {T_{r1} = t_{3} - t_{2} } \\ {T_{r2} = t_{5} - t_{4} } \\ \end{array} } \right. $$

2 $$ \hat{T}_{p} = (T_{R1} - T_{r1} )/2 = (T_{R2} - T_{r2} )/2 $$

The transformation of Eq. (2) yields:

3 $$ \left\{ {\begin{array}{*{20}c} {T_{R1} = 2\hat{T}_{p} + T_{r1} } \\ {T_{R2} = 2\hat{T}_{p} + T_{r2} } \\ \end{array} } \right. $$

Then we can get $T_{R1} *T_{R2} = (2\hat{T}_{p} + T_{r1} )*(2\hat{T}_{p} + T_{r2} ) = 4*\hat{T}_{p}^{2} + 2\hat{T}_{p} T_{r2} + 2\hat{T}_{p} T_{r1} + T_{r1} T_{r2}$.

which then yields $T_{R1} *T_{R2} - T_{r1} T_{r2} = 4*\hat{T}_{p}^{2} + 2\hat{T}_{p} T_{r2} + 2\hat{T}_{p} T_{r1} = \hat{T}_{p} (4\hat{T}_{p} + 2T_{r1} + 2T_{r2} )$.

According to (2) $4\hat{T}_{p} = T_{R1} - T_{r1} + T_{R2} - T_{r2}$ can be brought into the above equation to get:

4 $$ T_{R1} *T_{R2} - T_{r1} T_{r2} = \hat{T}_{p} (T_{R1} + T_{R2} + T_{r1} + T_{r2} ) $$

5 $$ \hat{T}_{{\text{p}}} = \frac{{(T_{R1} \times T_{R2} - T_{r1} \times T_{r2} )}}{{(T_{R1} + T_{R2} + T_{r1} + T_{r2} )}} $$

The distance from device A to device B is:

6 $$ D_{AB} = c*\hat{T}_{p} $$

where $c$ denotes the speed of light.

The formula of DS-TWR ranging error calculation is:

7 $$ error = \hat{T}_{p} \times (1 - \frac{{k_{a} + k_{b} }}{2}) $$

where $k_{a}$ and $k_{b}$ denote the frequencies of device A and device B, respectively.

UWB ranging has many advantages, such as centimeter-level high accuracy, high resolution, anti-interference, applicability indoors and outdoors, and low power consumption.

### Long short-term memory network fused with Kalman filter

#### Long short-term memory network

**Figure 2:**
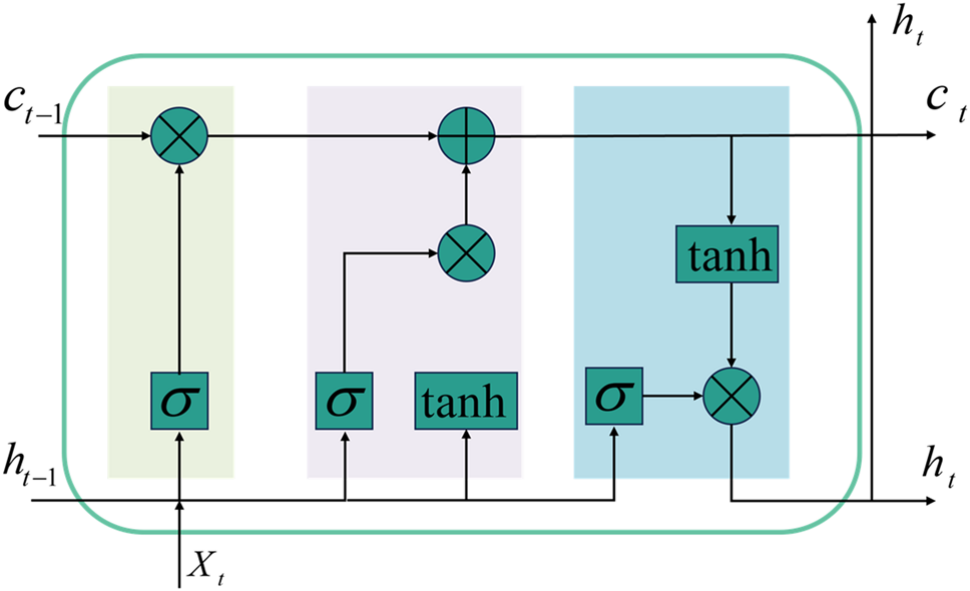
The basic structure of the LSTM network.

LSTM network evolves from the recurrent neural network (RNN), which solves the vanishing gradient problem in RNN^37^. The LSTM network has an additional time dimension in the input compared with general neural networks, such as the BP neural network, and it has a feedback loop, which can establish a connection between input data and better process time-related data. The core of LSTM is to introduce “gates” to control the flow of information. Each gate is a neural network layer that can control whether information can pass through; its main components include the input gate, forget gate, output gate, and cell state. Figure 2 shows the basic structure of the LSTM network:

The calculation of the input gate is:

8 $$ i_{t} = \sigma (W_{xi} x_{t} + W_{hi} h_{t - 1} + W_{ci} c_{t - 1} + b_{i} ) $$

where $i_{t}$ represents the input gate, $x_{t}$ represents the input at the current time, $h_{t - 1}$ represents the hidden state at the previous moment, and $c_{t - 1}$ represents the memory unit state at the previous moment. $W_{xi}$, $W_{hi}$, and $W_{ci}$ are weight matrices, $b_{i}$ is the bias vector, and $\sigma$ is the sigmoid activation function.

The calculation of the forgetting gate is:

9 $$ f_{t} = \sigma (W_{xf} x_{t} + W_{hf} h_{t - 1} + W_{cf} c_{t - 1} + b_{f} ) $$

The forget gate determines how much information is retained in the memory unit state at the previous time step, where $W_{xf}$, $W_{hf}$, and $W_{cf}$ are the weight matrices, and $b_{f}$ is the bias vector.

The memory unit state is updated as:

10 $$ \tilde{c} = \tanh (W_{xc} x_{t} + W_{hc} h_{t - 1} + b_{c} ) $$

11 $$ c_{t} = f_{t} \odot c_{t - 1} + i_{t} \odot \tilde{c}_{t} $$

where tanh represents the tanh activation function; $\tilde{c}$ represents the candidate memory unit state of the current time step, indicating the state of the memory unit at the current time step. $W_{cx}$ and $W_{hc}$ represent the weight matrix input for updating the memory unit state. $b_{c}$ represents the bias term for updating the memory unit state. $\odot$ represents element-wise product, i.e., multiplying elements at corresponding positions.

The calculation of the output gate is:

12 $$ o_{t} = \sigma (W_{xo} x_{t} + W_{ho} h_{t - 1} + W_{co} c_{t} + b_{o} ) $$

The output gate determines the output of the hidden state at the current time step. $o_{t}$ represents the output gate; $W_{xo}$, $W_{ho}$, and $W_{co}$ are weights; $b_{o}$ is the bias vector.

The hidden state at the current moment is calculated as:

13 $$ h_{t} = o_{t} \odot \tanh (c_{t} ) $$

### Kalman filter

KF is a linear estimation algorithm suitable for solving linear problems with Gaussian white noise. By fusing the system model and the observation model, it obtains the past state estimates and the latest measured values based on the minimum mean. The square root error criterion is used for weighted estimation to obtain the optimal estimate of the system^38^. This study uses the Kalman filter to filter the time series of LSTM to make the results more accurate.

The KF algorithm is divided into two steps: prediction and update. The time update equation of the KF is as follows:

14 $$ \left\{ {\begin{array}{*{20}c} {\hat{x}_{{\overline{k}}} = A\hat{x}_{k - 1} + Bu_{k - 1} } \\ {P_{{\overline{k}}} = AP_{k - 1} A^{T} + Q} \\ \end{array} } \right. $$

where $\hat{x}_{{\overline{k}}}$ represents the prior state estimate at time $k$. $\hat{x}_{k - 1}$ represents the state estimate at time $k - 1$. A is the state transition matrix, and B is the matrix that converts the input into a state. $\mu_{k - 1}$ represents the control quantity at moment $k - 1$.$P_{{\overline{k}}}$ represents the estimated covariance of $\hat{x}_{{\overline{k}}}$ at time $k$. $P_{k - 1}$ represents the estimated covariance at time $k - 1$. The matrix $Q$ is the process excitation noise covariance. The first equation in Formula (14) represents the prior state estimate, while the second equation calculates the covariance of the prior estimate.

The state update equation of the Kalman filter is as follows:

15 $$ \left\{ {\begin{array}{*{20}c} {\hat{x}_{k} = \hat{x}_{{\overline{k}}} + K_{k} (z_{k} - H\hat{x}_{{\overline{k}}} )} \\ {K_{k} = \frac{{P_{{\overline{k}}} H^{T} }}{{HP_{{\overline{k}}} H^{T} + R}}} \\ {P_{k} = (I - K_{k} H)P_{{\overline{k}}} } \\ \end{array} } \right. $$

where $\hat{x}_{k}$ represents the state estimate at time $k$. $P_{k}$ represents the estimated covariance at time $k$. The matrix $H$ is the state-to-observation transition matrix measurement.$z_{k}$ is the measured value. $K_{k}$ is the Kalman gain matrix. $R$ represents the measurement noise covariance. The first equation in Formula (15) is the posterior state estimate, the second equation is the update of the Kalman gain, and the third equation is the final covariance calculation.

## UWB positioning based on the KF–LSTM algorithm

**Figure 3:**
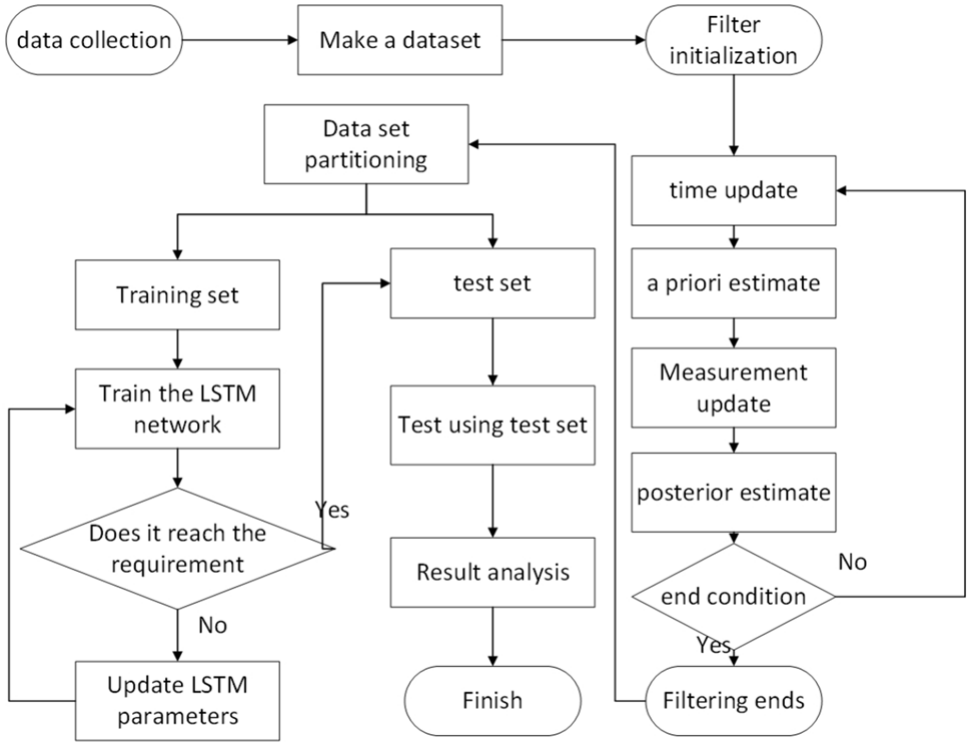
The flow chart of the KF-LSTM algorithm.

The data and time used for positioning using UWB ranging are related. The LSTM network is a deep learning model for processing time series, and it can extract dependencies in time series. The Kalman filter is an optimal linear filter that obtains the best state estimate based on time series and state transition models. In practical applications, experimental data contain noise, and there is uncertainty in the state of the system. Kalman filtering can effectively handle these noises and uncertainties, providing more accurate state estimates for experiments. The combination of the Kalman filter and the LSTM network can simultaneously exploit the LSTM’s advantage in timing modeling and the Kalman filter’s advantage in state estimation, thereby providing more accurate results for experiments. The algorithm is divided into three steps: establishing a time series data set, establishing a Kalman filter equation, and building an LSTM neural network. The specific process is shown in Fig. 3.

The number of UWB base stations used in the experiment is $N$, and under ideal circumstances, $N \ge 3$ can uniquely determine the location of the tag. If there is only one tag in the UWB positioning system, the distance from the tag to each base station at time $t$ is recorded as $$\left[ {\begin{array}{*{20}c} {D^{t}_{1} } & {D^{t}_{2} } & {D^{t}_{3} } & {\begin{array}{*{20}c} {...} & {D^{t}_{N} } \\ \end{array} } \\ \end{array} } \right]$$. If the length of the time series required to construct the data set is $T$, then a piece of data in the data set can be recorded as:

16 $$ \left[ {\begin{array}{*{20}c} {D_{1}^{1} } & {D_{2}^{1} } & {D_{3}^{1} } & {\begin{array}{*{20}c} {...} & {D_{N}^{1} } \\ \end{array} } \\ {D_{1}^{2} } & {D_{2}^{2} } & {D_{3}^{2} } & {\begin{array}{*{20}c} {...} & {D_{N}^{2} } \\ \end{array} } \\ {D_{1}^{3} } & {D_{2}^{3} } & {D_{D}^{3} } & {\begin{array}{*{20}c} {...} & {D_{N}^{3} } \\ \end{array} } \\ {\begin{array}{*{20}c} \vdots \\ {D_{1}^{T} } \\ \end{array} } & {\begin{array}{*{20}c} \vdots \\ {D_{2}^{T} } \\ \end{array} } & {\begin{array}{*{20}c} \vdots \\ {D_{3}^{T} } \\ \end{array} } & {\begin{array}{*{20}c} {\begin{array}{*{20}c} \ddots \\ \ldots \\ \end{array} } & {\begin{array}{*{20}c} \vdots \\ {D_{N}^{T} } \\ \end{array} } \\ \end{array} } \\ \end{array} } \right] $$

Each column of data represents the distance measured between a base station and the tag within time $T$. Each column of data is subjected to a Kalman filter to reduce the noise and uncertainty of the data. The state equation and observation equation of the Kalman filter are represented as follows:

17 $$ \left\{ {\begin{array}{*{20}c} {\hat{D}_{i}^{t} = \hat{D}_{i}^{t - 1} + u_{i}^{t} } \\ {z_{i}^{t} = \hat{D}_{i}^{t} + v_{i}^{t} } \\ \end{array} } \right. $$

where $\hat{D}_{i}^{t - 1}$ and $\hat{D}_{i}^{t}$ represent the estimated values of the base station and tag at time $t - 1$ and $t$, respectively. $z_{i}^{t}$ represents the observation value of the i-th base station and tag at time $t$. $u_{i}^{t}$ and $v_{i}^{t}$ represent the estimation error and observation error of the i-th base station and tag at time $t$.

The state update formula of the Kalman filter is as follows:

18 $$ \left\{ {\begin{array}{*{20}c} {\hat{D}_{i}^{t} = \hat{D}_{i}^{t - 1} + K_{t} (z_{i}^{t} - \hat{D}_{i}^{t - 1} )} \\ {K_{t} = \frac{{u_{i}^{t} }}{{u_{i}^{t} + v_{i}^{t} }}} \\ {u_{i}^{t} = (1 - K_{t} )u_{i}^{t} } \\ \end{array} } \right. $$

where $K_{t}$ is the Kalman gain coefficient at time $t$.

Since the distance from the base station to the tag cannot be estimated empirically, the first measured value is selected as the initial estimated value. The LSTM network is constructed based on Python 3.7 and Tensorflow 1.8. The size of the input layer is 4*10, the number of nodes in the hidden layer is set to 64, the number of layers in the network is set to 2, and the loss function uses the root mean square error function. The error loss function is as follows:

19 $$ loss = \frac{{(\sum\limits_{i = 1}^{n} {(x\_p - x)^{2} + } \sum\limits_{i = 1}^{n} {(y\_p - y)^{2} } )}}{n} $$

where $loss$ denotes the loss value; $x\_p$ and $y\_p$ denote the coordinate prediction values calculated by the LSTM network; $x$ and $y$ denote the true values of the coordinates; $n$ denotes the amount of data in the training data set.

Meanwhile, an exponential decay function is selected for the learning rate. After a certain number of rounds of training, the learning rate decays at a certain rate. This approach can help the model converge faster in the early stages, and the gradually reduced learning rate also improves the stability and generalization ability of the model.

## Data simulation and result analysis

### Data simulation

To verify the theoretical feasibility of the proposed algorithm, this study uses Python 3.7 software to conduct simulation experiments and result analysis. In the simulation experiment, four UWB base stations were used, marked A, B, C, and D respectively, and their coordinates were recorded as $(x_{A} ,y_{A} )$, $(x_{B} ,y_{B} )$, $(x_{C} ,y_{C} )$, and $(x_{D} ,y_{D} )$. Also, it is necessary to record the coordinates of label E $(x_{E} ,y_{E} )$. The real distance from A to E is:

20 $$ d_{AE} = \sqrt {(x_{A} - x_{E} )^{2} + (y_{A} - y_{E} )^{2} } $$

In the simulation experiment, the coordinates of the base station were set as (0,0) (0,400) (400,400), and (400,0), and the coordinate unit was centimeters. During the experiment, a circular route containing 1000 points was generated in the experimental area. To effectively train and verify the algorithm, this data set was divided into a training set and a verification set at a ratio of 10:1, i.e., the training set contained 90% of the data points (900 points), while the validation set contained 10% of the data points (100 points). The real distance between these points and the base station was obtained following Eq. (16), and a certain amount of noise was added to the generated real distance to obtain the LSTM time series required for research. To simulate the complex and changeable indoor environment and verify the noise suppression effect of the KF–LSTM algorithm, ten types of noise were set in the simulation data. The standard deviation of the noise from small to large is 10 cm, 20 cm, 30 cm, 40 cm, 50 cm, 60 cm, 70 cm, 80 cm, 90 cm, and 100 cm. The data set constructed in the experiment had a time step of 10, and the measured distances of the four base stations corresponded to four features.

### Simulation result analysis

**Figure 4:**
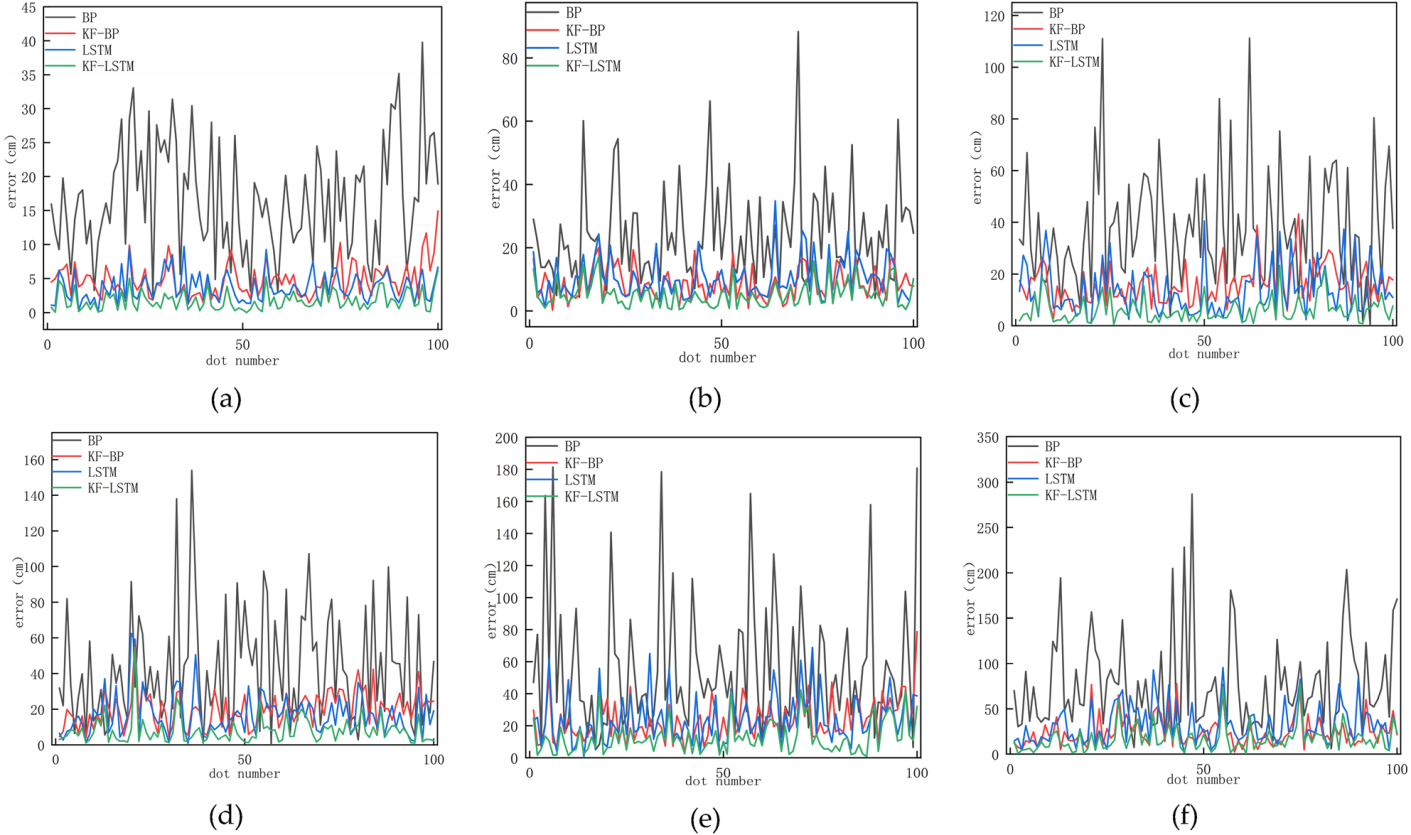
The error curves for the four algorithms’ calculation results under the noise standard deviation of 10 cm, 30 cm, 50 cm, 60 cm, 80 cm, and 100 cm are displayed in (**a**), (**b**), (**c**), (**d**), (**e**), and (**f**), in that order.

**Figure 5:**
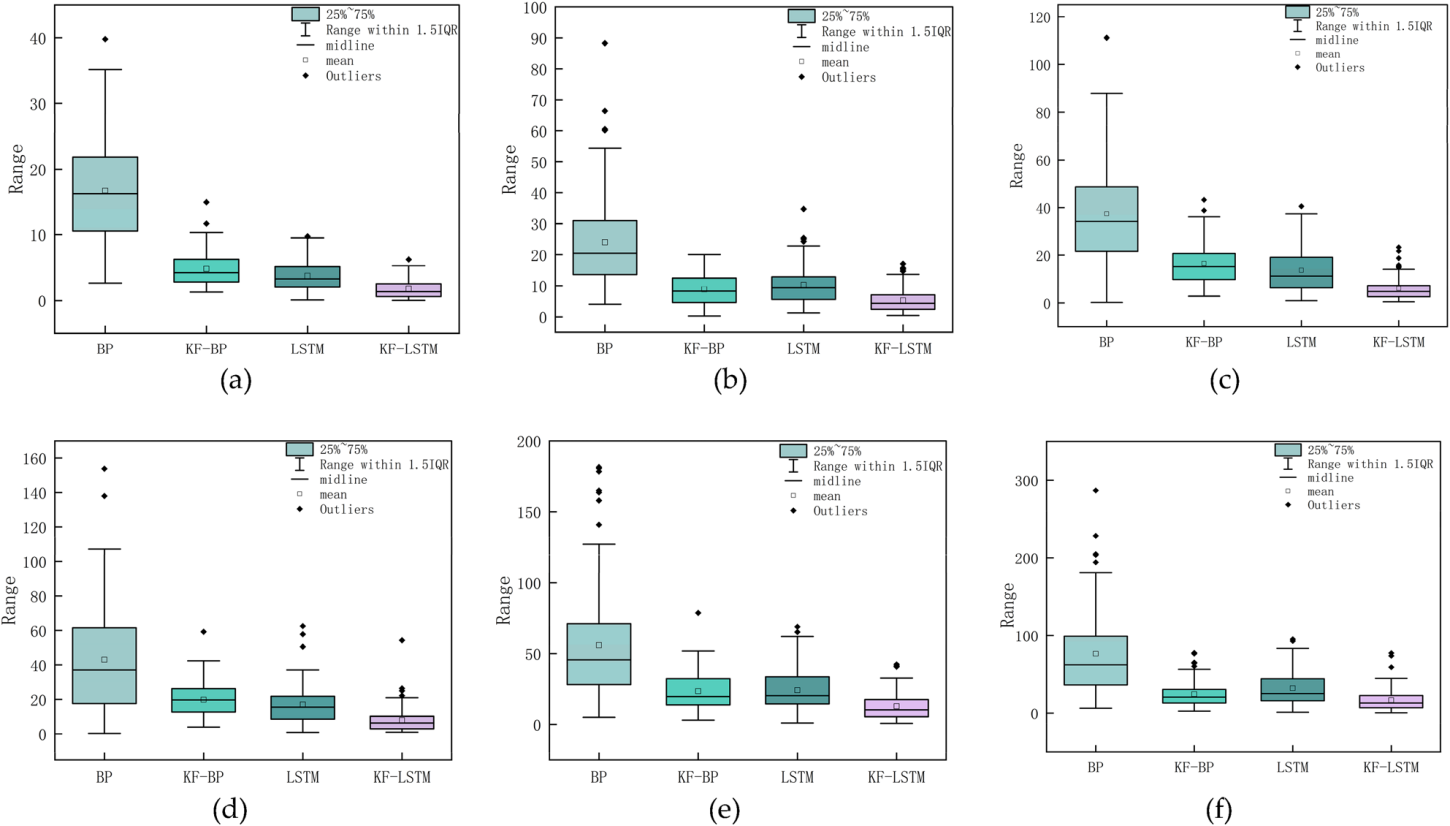
When the noise standard deviation is 10 cm, 30 cm, 50 cm, 60 cm, 80 cm, and 100 cm, the box plots of the four algorithms are displayed in (**a**), (**b**), (**c**), (**d**), (**e**), and (**f**), in that order.

Figure 4 shows that regardless of the type of noise, the overall error of the BP algorithm is the largest, while the overall error of the KF–LSTM algorithm is the smallest. Next are the KF-BP and LSTM algorithms, where the precision of the BP algorithm is the lowest. To show the overall error status of the four algorithms under different noise conditions, the box plots of the four algorithms under different noises are illustrated in Fig. 5.

**Figure 6:**
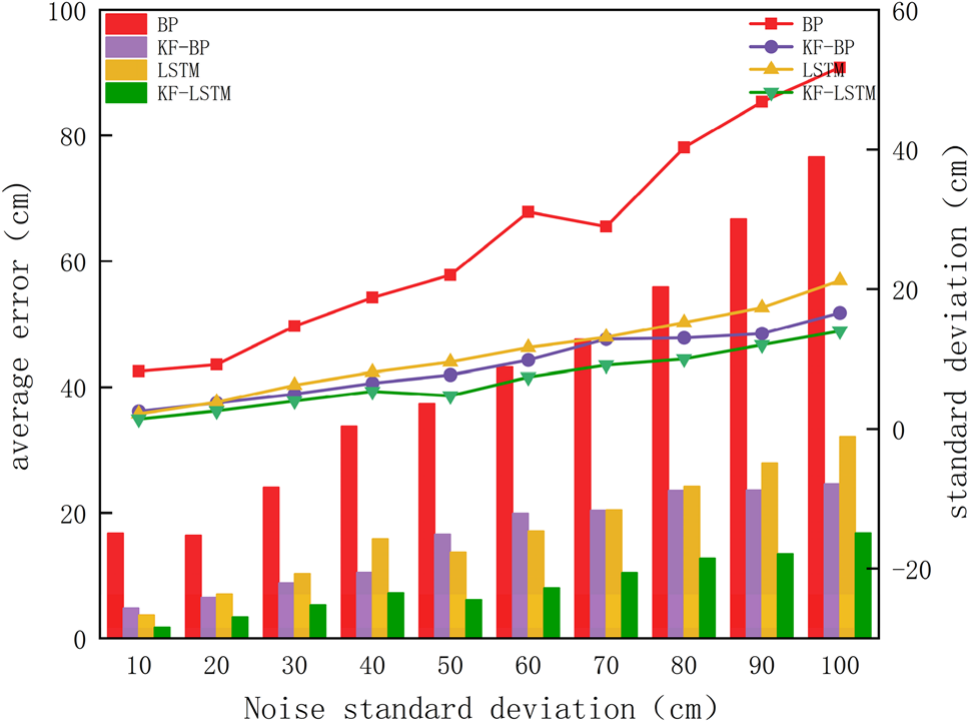
The average error and standard deviation of the four algorithms.

Figure 5 shows that the average error of each algorithm increases as the noise standard deviation increases. Regardless of the type of noise, the BP algorithm has the largest box, the widest error range, and the largest average error. The KF–LSTM algorithm has the smallest box, the smallest error distribution range, and the smallest average error. To illustrate how the accuracy and stability of the four algorithms change with noise, Fig. 6 shows the changes in the average positioning error and standard deviation under different noises.

In Fig. 6, the histogram display corresponds to the left axis, showing the mean error for each algorithm, and the line graph corresponds to the right axis, showing the standard deviation for each algorithm. Where the mean error responds to the accuracy of the algorithm and the standard deviation responds to the stability of the algorithm. Among the four algorithms, the accuracy and standard deviation of the BP algorithm are the worst under various noises. However, incorporating Kalman filtering into the BP algorithm (KF-BP) greatly improves the accuracy and stability of the algorithm. In multiple datasets with a noise standard deviation below 70 cm, it is not possible to determine a consistent superiority or inferiority in terms of accuracy between the KF-BP algorithm and the LSTM algorithm. However, when the noise standard deviation in the dataset exceeds 70 cm, the average error of the KF-BP algorithm is consistently lower than that of the LSTM algorithm. Furthermore, in terms of stability analysis, when the dataset includes noise with a standard deviation higher than 20 cm, the stability of the KF-BP algorithm is superior to that of the LSTM algorithm. This also explains why KF–LSTM exhibits the best accuracy and stability under any level of noise. Combined with Fig. 5, it can be concluded that the KF–LSTM algorithm has better positioning accuracy and stability than the other three algorithms. Taking the noise standard deviation of 30 cm as an example, the KF–LSTM algorithm has 78.05% higher accuracy than the BP algorithm, 40.33% higher accuracy than the KF-BP algorithm, and 48.73% higher accuracy than the LSTM algorithm. The specific values of the average error and standard deviation of various algorithms under different noises are listed in Table 1.

**Table 1:** The average error and standard deviation of the four algorithms under different noise.

Noise standard deviation (cm)	Average error (cm)				Standard deviation(cm)			
	BP	KF-BP	LSTM	KF-LSTM	BP	KF-BP	LSTM	KF-LSTM
10	16.7	4.8	3.7	1.8	8.2	2.5	2.1	1.4
20	16.3	6.5	7.1	3.4	9.2	3.7	3.8	2.6
30	24	8.8	10.3	5.3	14.7	5	6.3	4
40	33.8	10.5	15.8	7.3	18.7	6.6	8.1	5.4
50	37.4	16.6	13.7	6.1	22	7.7	9.6	4.8
60	43.2	19.9	17	8	31	9.8	11.6	7.4
70	47.6	20.4	20.5	10.5	29	12.8	13.1	9.1
80	55.9	23.5	24.2	12.8	40.3	13	15.2	10
90	66.7	23.6	27.9	13.5	46.9	13.7	177.3	12
100	76.6	24.6	32	16.8	51.8	16.6	21.2	14

## Measured data and result analysis

### Data collection

**Figure 7:**
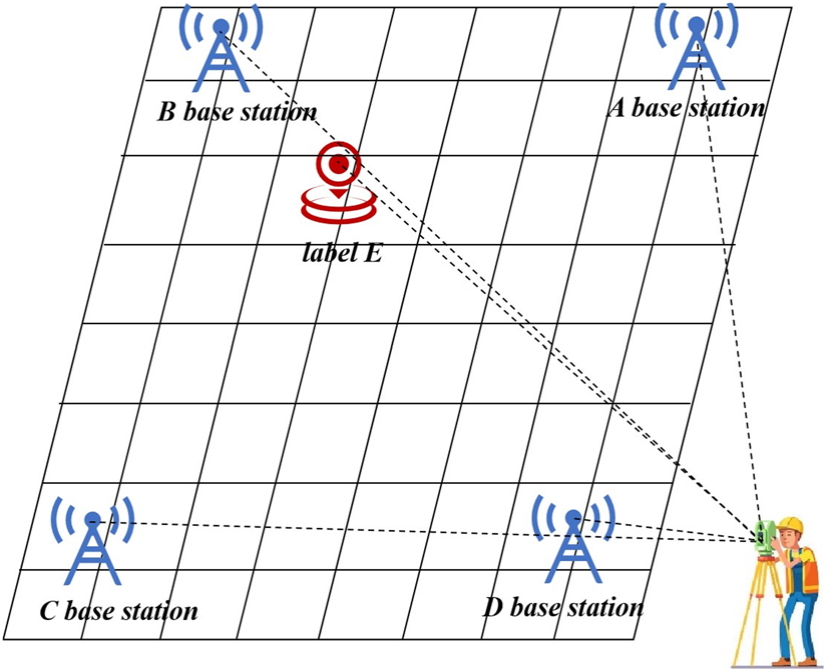
The schematic diagram of the experimental site.

Then, this study conducts actual experiments to verify the feasibility of the proposed algorithm in practical applications. The experimental site was chosen at the School of Surveying and Mapping of Henan Polytechnic University, with a site size of 8 m × 8 m. In our experiment, the coverage area of base station is 6 m × 6 m. The experiment used a UWB device with the DW1000 chip as the label and tracing point. The ranging principle of the device is based on DS-TWR. The experiment selected four UWB base stations and one UWB tag. In the experiment, a total station was selected as an auxiliary to measure the real coordinates of the base station and tag. The positions of the base station erection position and total station are shown in Fig. 7. The coordinates of base station A in Fig. 7 are (263.9, 676), base station B is located at (837, 562.4), base station C at (774, 88.7), and base station D at (193.2, 67.7), with units in centimeters.

### System error handling

**Figure 8:**
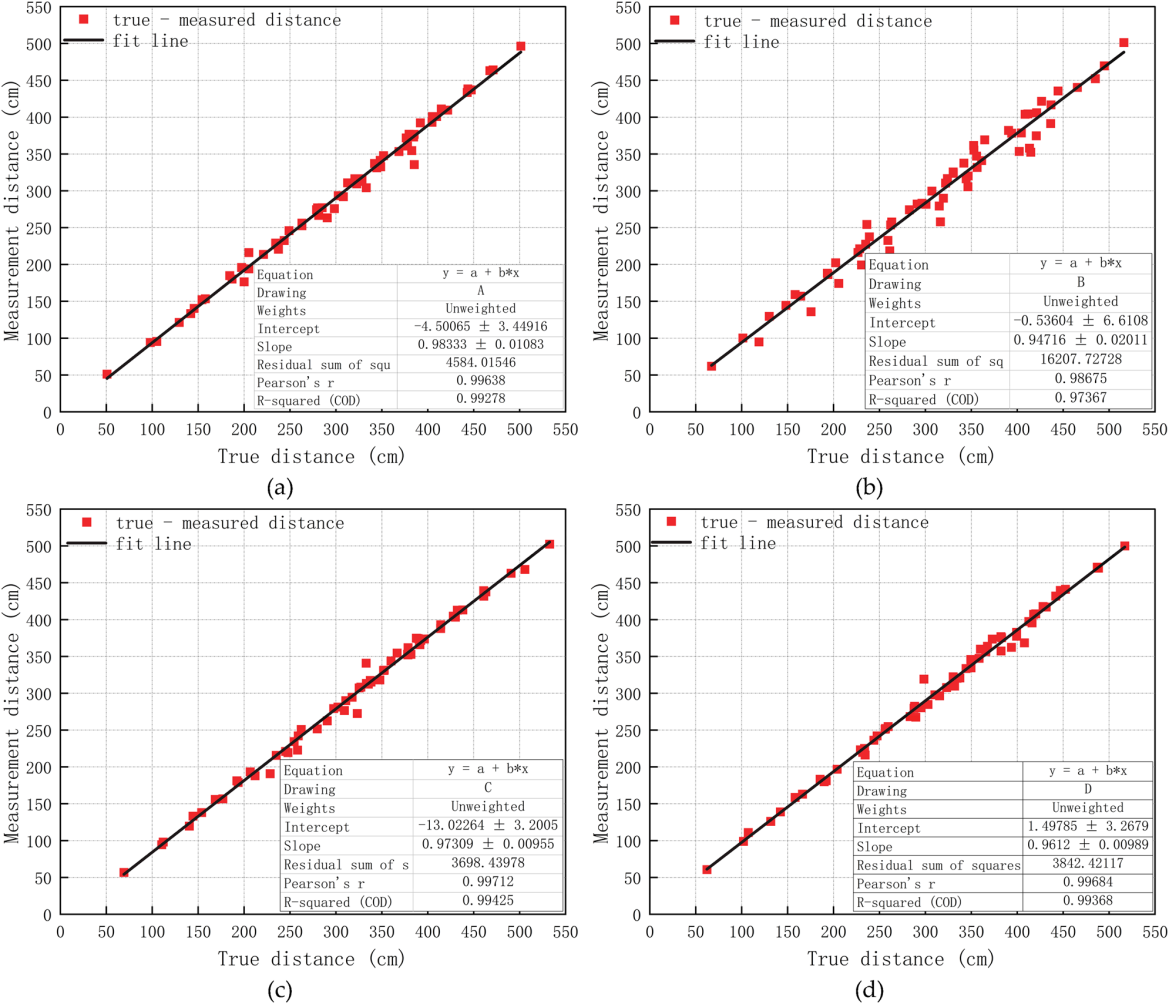
(**a**), (**b**), (**c**), and (**d**) show the least squares fitting results of base stations A, B, C, and D, respectively.

Since there are systematic errors in the measured values, there are no systematic errors in the simulation experiments. To make the calculation results more accurate, the systematic errors need to be weakened. To this end, the least squares criterion is adopted to establish a linear regression equation for each base station. The relevant parameters of the equation are listed in Table 2. The fitting situations of base stations A, B, C, and D are shown in Fig. 8. The system errors of base stations A, B, C, and D are 4.5 cm, 0.5 cm, 13 cm, and 1.5 cm, respectively. After removing the systematic error, the obtained results are sent to the KF–LSTM algorithm for solution.

**Table 2:** Coefficients and Offset for linear fitting of four base stations.

Base station	Coefficient (cm)	Offset (cm)
A	0.98	4.5
B	0.94	0.5
C	0.97	13
D	0.96	1.5

### Result analysis

**Figure 9:**
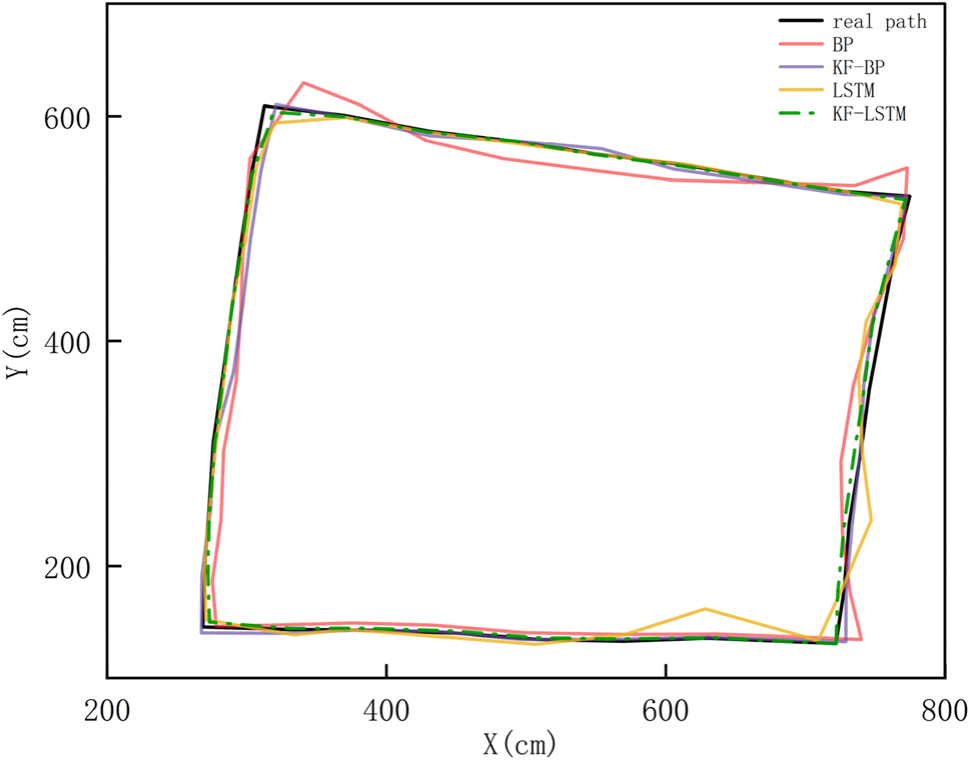
The roadmap calculated by the four algorithms.

The field measurement data after weakening the impact of system errors are brought into the KF–LSTM network for iterative operations. The results of the BP, KF-BP, LSTM, and KF–LSTM algorithms are compared. Figure 9 shows the original path and the path map calculated by the KF–LSTM, BP, KF-BP, and LSTM algorithms. It can be seen from the figure that the road map of the KF–LSTM algorithm is closest to the original path, followed by KF-BP and LSTM algorithms, while the road map of the BP algorithm is the worst and least close to the original path.

**Figure 10:**
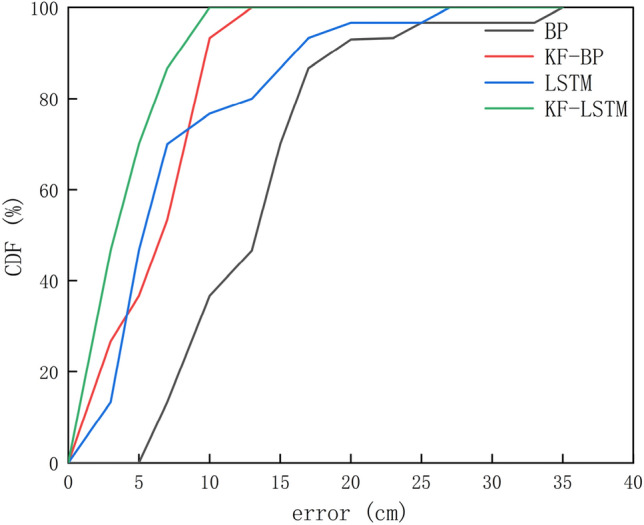
The CDF curve.

**Figure 11:**
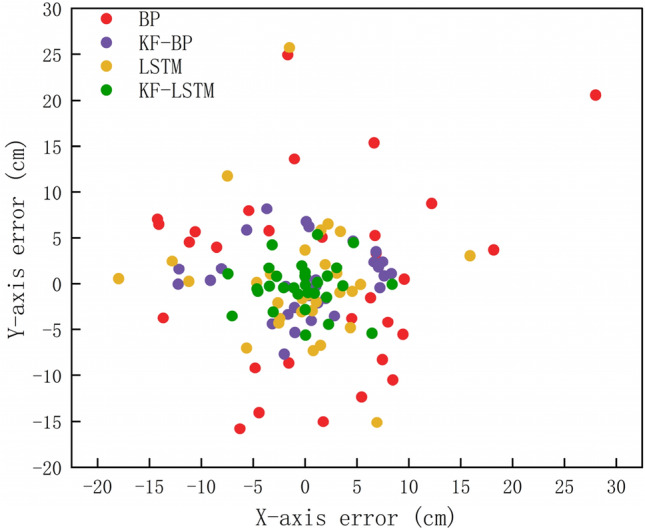
Error distribution.

Figure 10 shows the error cumulative distribution function (CDF) of various algorithms. It can be seen that under the same threshold, the KF–LSTM algorithm obtains the highest positioning error probability. The distance error is within 7 cm, the cumulative probabilities corresponding to the BP, KF-BP, LSTM, and KF–LSTM algorithms are 13.33%, 53.33%, 70%, and 86.67% respectively. It can be seen that the positioning error range of the KF-LSTM algorithm is better than those of the other three algorithms. The error distribution of the four methods is displayed in Fig. 11. The distribution of the BP and LSTM algorithms is relatively scattered, i.e., the stability of the BP algorithm and the LSTM algorithm is not as good as that of the KF-BP and KF-LSTM algorithms. The most concentrated results near the (0,0) point are those of the KF-LSTM algorithm, so the KF-LSTM algorithm achieves the highest accuracy.

**Figure 12:**
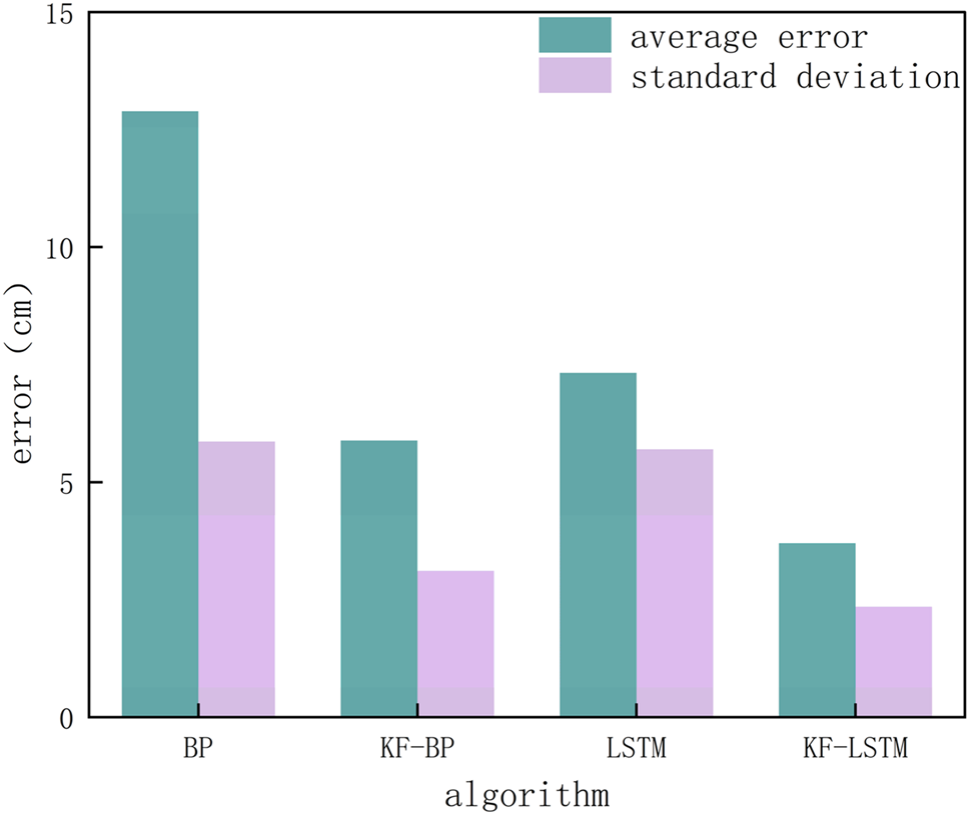
The average error and standard deviation of the four algorithms.

The four algorithms’ average error and standard deviation are displayed in Fig. 12. The average error of the KF-LSTM algorithm is 3.7 cm, and those of the BP, KF-BP, and LSTM algorithms are 12.9 cm, 5.9 cm, and 7.3 cm, respectively. In terms of accuracy, the KF-LSTM algorithm achieves the highest accuracy, which is 71.31%, 37.28%, and 49.31% higher than that of the BP, KF-BP, and LSTM algorithms respectively. From the perspective of stability analysis, the standard deviations of the BP, KF-BP, LSTM, and KF-LSTM algorithms are 5.86 cm, 3.11 cm, 5.7 cm, and 2.35 cm, respectively. The standard deviation of the KF-LSTM algorithm is the smallest, so the KF-LSTM algorithm has the best stability.

## Discussion

UWB has good prospects in the field of indoor localization, but noise interferences often occur during the propagation of UWB signals. These interferences will affect the accuracy of UWB ranging, which will in turn affect the positioning accuracy of UWB. To weaken the influence of noise and improve the positioning accuracy of UWB, this paper proposes the KF-LSTM algorithm. And confirmed that the KF-LSTM algorithm could be used for indoor localization.

The paper proposes the KF-LSTM algorithm by combining the advantages of Kalman filtering and LSTM neural networks to improve the accuracy of UWB positioning. The Kalman filter can assist the LSTM algorithm in handling errors and uncertainties in UWB ranging. To verify the degree of noise suppression of the proposed algorithm, different levels of noise are added to the simulation data. Simulation results show that the algorithm incorporating Kalman filtering improves both accuracy and stability, and the effect is more pronounced the larger the standard deviation of the noise included in the data. Later, considering the impact of systematic errors in actual experiments, least squares fitting was used to weaken the systematic errors. The LSTM algorithm can process sequence data and dependencies in UWB positioning, provide nonlinear modeling, and cooperate with Kalman filtering to better adapt to dynamic environments and improve the accuracy of UWB positioning. The real experiments also show that the KF-LSTM algorithm inherits the advantages of the Kalman filter and LSTM and outperforms the other three algorithms in terms of accuracy and stability, which is consistent with the results obtained from simulation.

However, the KF-LSTM algorithm still has defects. For instance, the DWM1000 ranging frequency is 100 HZ, and the algorithm requires ten distances to position each base station for one positioning, i.e., theoretically, positioning can be given in 0.1 s, so the algorithm is only suitable for low-rate applications when performing indoor positioning. Moreover, the time sequence required by the KF-LSTM algorithm is fixed, so if any base station loses contact during positioning, there will be positioning errors.

UWB has demonstrated excellent positioning accuracy in specific environments. However, considering the high complexity and uncertainty of indoor environments, UWB still faces huge challenges in its widespread application for indoor positioning. In the future, we will optimize this algorithm and consider fusing multiple LSTM models to meet various data input conditions to deal with the loss of connection of base stations and partial data loss.

## Conclusions

This paper proposes an LSTM neural network algorithm fused with the Kalman filter for UWB indoor positioning. This algorithm transforms the distance data measured by the UWB device into a time series, then uses Kalman filtering to process the noise and uncertainty factors in the time series, and finally trains the processed time series in the LSTM network. In the simulation experiment, the KF-LSTM algorithm achieves higher positioning accuracy and stability than the BP, KF-BP, and LSTM algorithms under any noise. The greater the noise in the data, the more obvious the contrast in accuracy and stability of the four algorithms. In the actual measurement experiment, the average positioning accuracy of the KF-LSTM algorithm reaches 3.7 cm, showing an improvement of 71.31%, 37.28%, and 49.31% respectively compared with the BP, KF-BP, and LSTM algorithms. Moreover, the standard deviation of the KF-LSTM algorithm is only 2.35 cm, while the standard deviations of the BP, KF-BP, and LSTM algorithms are 5.86 cm, 3.11 cm, and 5.7 cm, respectively. Therefore, the KF-LSTM algorithm is the most stable among the four algorithms, which is consistent with the results of the simulation experiment.

## Data Availability

These were computer-generated and gathered in the experimental area; they are not yet accessible to the general public or over the internet. If necessary, they can be acquired from the corresponding author.
